# Characterizing implementation strategies using a systems engineering survey and interview tool: a comparison across 10 prevention programs for drug abuse and HIV sexual risk behavior

**DOI:** 10.1186/s13012-016-0433-3

**Published:** 2016-05-17

**Authors:** Sara J. Czaja, Thomas W. Valente, Sankaran N. Nair, Juan A. Villamar, C. Hendricks Brown

**Affiliations:** 1 Department of Psychiatry and Behavioral Science, Miller School of Medicine, University of Miami, 1694 NW 9th Ave., Miami, FL 33136 USA; 2 Department of Preventive Medicine, Keck School of Medicine, University of Southern California, Los Angeles, USA; 3 Center for Aging, Miller School of Medicine, University of Miami, Miami, USA; 4 Department of Psychiatry and Behavioral Sciences, Feinberg School of Medicine, Northwestern University, Evanston, USA

**Keywords:** Implementation science, Behavioral interventions, Systems engineering

## Abstract

**Background:**

Although many behavioral interventions have proven to be efficacious, new methodologies are required beyond efficacy trials to understand how to adopt, implement with fidelity, and sustain behavioral interventions in community settings. In this paper, we present a new approach, based on systems engineering concepts and methods, for characterizing implementation strategies that are used to deliver evidence-based behavioral interventions in health and social service settings. We demonstrate the use of this approach with implementation strategies, used or being used for broader dissemination of 10 evidence-based prevention program projects focused on the prevention of drug or HIV sex risk behaviors.

**Results:**

The results indicate that there are wide variations in intervention approaches and that there are challenges in program implementation including maintaining program fidelity, serving community needs, and adequate resources. The results also indicate that implementation requires a committed partnership between the program developers, implementation researchers, and community partners. In addition, there is a need for adaptability within programs to meet community needs, resources, and priorities while maintaining program fidelity.

**Conclusions:**

Our methodological approach enabled us to highlight challenges associated with the community implementation of health risk prevention interventions. We also demonstrate how comprehensive descriptions of interventions facilitate understanding of the requirements of program implementation and decisions about the feasibility of implementing a program in community settings.

## Background

Drug use and sexually transmitted infections remain significant public health problems in the USA. In 2011, an estimated 8.7 % of the population had used or abused an illicit drug or a psychotherapeutic medication; of these 24 % were 18–20 year olds [1]. Drug and alcohol disorders and sexually risky behaviors, which increase the chance of sexually transmitted infections, are closely tied especially in adolescence [1–8].

In this regard, a large number of interventions have been shown to be effective in reducing the incidence of drug and alcohol abuse [9] and engagement in risky sexual behavior among young adults (e.g., [10, 11]). But there are challenges to widely delivering these programs [12], especially to minority populations [13, 14]. Despite the recent “policy push” to promote community-based adoption of evidence-based prevention programs, adoption is painstakingly slow and there is a tension between maintaining intervention fidelity while meeting community values, needs, and constraints. Overall, although there is a relatively robust body of research on effective interventions, the adoption of these interventions in clinical and community practice has lagged behind.

**Fig. 1:**
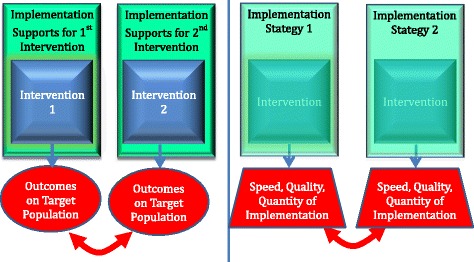
Comparison of an effectiveness trial (*left*) of two interventions with an implementation trial (*Right*) of one intervention delivered through two implementation strategies

To that end, research is required beyond efficacy and effectiveness trials to understand how to adopt, implement with fidelity, and sustain behavioral interventions in community settings [15]. Although significant research is being conducted in this area [13, 16], the field of implementation science is still in its infancy [17]. Progress requires the development of novel, rigorous designs to test alternative strategies to delivering evidence-based programs. Randomized trials in which organizations and communities are assigned randomly to different implementation strategies have been recommended [18, 19] and are starting to be implemented [18]. Figure 1 compares the differences between an effectiveness trial that tests the impact of an intervention on a target population’s outcome (e.g., drug use) (left side of the figure) and that of an implementation trial where an intervention is administered through two different implementation strategies (right side of the figure). In the intervention trial, two intervention strategies are in the foreground and in the background are the system level supports for delivering the interventions; the primary outcome is directed at the target population. In the implementation trial, two implementation strategies are in the foreground and a single intervention is in the background. The primary outcomes are related to intervention delivery [18, 20]. With the specification of objective measures of the implementation process, such as the Stages of Implementation Completion (SIC) [21, 22], implementation trials show promise in helping us learn what approaches are most successful in implementing evidence-based interventions [13, 23].

However, two major challenges remain; a limited shared language to describe interventions/implementation approaches and tools to characterize the goals and specific elements of intervention/implementation strategies. This paper describes one set of tools, based on a systems engineering approach, which can be used to elucidate the resources and other requirements necessary for successful implementation of an intervention in a consistent way. The tools are applied to characterize and compare implementation strategies that have been or will be tested through randomized implementation trials for broad dissemination of 10 evidence-based prevention programs focused on drug abuse and/or risky sexual behaviors. We begin with a brief overview of the 10 programs followed by an overview of systems engineering.

## Methods

### Overview of the 10 evidence-based programs

Among the 10 evidence-based prevention programs included in this study, some focused on drugs or sexual risk only, others focused on both, and still others, which targeted risk and protective factors at birth or in early elementary school, focused on important antecedent factors in these earlier stages of life before entering the period of high risk. Because of the high frequency of drug and sex risk behavior, as well as the overlap between these outcomes, programs that have demonstrated beneficial impact across these outcomes have great potential value if they can be implemented widely. The programs are all considered proven or promising evidence-based programs by either the Blueprints Project for Violence Prevention [24] or by the Centers for Disease Control and Prevention [25], and they constitute a network of leading prevention implementation researchers all affiliated with the National Institute on Drug Abuse’s funded Center for Prevention Implementation Methodology (Ce-PIM) for Drug Abuse and HIV Sexual Risk Behavior.

Table 1 presents general features of the 10 preventive intervention programs;^1^ many have undergone two or more decades of testing for efficacy and effectiveness and for some research has been or is being conducted on the program’s implementation strategy. Key references to both efficacy/effectiveness testing and implementation testing, when the latter exists, are provided.

**Table 1:** Summary of programs and implementation strategies

Prevention program name/Principal Investigator (references)	Program goals/significant impact	Stage of life	Implementation research and practice	Implementation agent(s)	Setting
Quit Using Drugs Intervention Trial (QUIT) Screening, brief intervention and referral for treatment (SBIRT)/Gelberg [57]	Prevent escalation of drug use to abuse/dependence for adults/drug use. The program has demonstrated effectiveness for reducing alcohol abuse and it is currently being tested on substance abuse [57]	Adulthood	Research: type 1 hybrid^a^ Practice: exploration phase^b^	Physician and health educator	Primary care clinics
Communities that Care: programs vary depending on community selection/Hawkins, Catalano [33]	Prevent youth externalizing behaviors and promote healthy development/violence, alcohol, drug, tobacco use	Childhood	Research: randomized implementation trial^a^ Practice: distributed widely through SAMHSA, sustainment^b^	Community coalition leaders	Rural, small towns and cities, and portions of metropolitan areas, including Latin America
Familias Unidas/Pantin, Prado [58]	Use parent training to increase parent-child communication/drug use, sex risk, depressive symptoms. Program reduced monthly substance use in Hispanics by 30 % and achieved higher frequency of condom use among adolescents sexually active 30 months after the intervention [26].	Middle school	Research: pilot type 3 hybrid^a^ Practice: exploration phase^b^	Caregivers, supported by trained facilitator	Small parent groups and home visits with family
Family check-up/Dishion, Stormshak [59]	Family management practices designed to reduce problem behaviors, enhance parenting skills, reduce family conflict, and reduce substance use. The program has demonstrated significant impact in preventing drug use [59–62] and depressive symptoms [63].	Elementary, middle, and high school	Research: randomized implementation trial^a^ Practice: active implementation^b^	Caregivers, supported by trained counselors	School settings and mental health community agencies
Good Behavior Game (GBG)/Poduska, Kellam [28]	Use group-based contingencies to reduce child aggressive disruptive behavior/drug and alcohol abuse or dependence disorder, conduct disorder, antisocial personality disorder, suicide ideation and attempts, criminal arrests, sex risk behavior. Results across 3 randomized control trials indicated a 50 % reduction in drug abuse/dependence disorders among males through age 21 [10, 64–69], a significant reduction in alcohol abuse/dependence disorders for males and females [10], and also demonstrated a reduction in unprotected and risky sexual behavior through young adulthood among the highest risk group [11].	First and second grade	Research: randomized implementation trial^a^ Practice: wide scale implementation through SAMHSA (http://www.samhsa.gov/grants/2010/sm-10-017.aspx); active implementation^b^	School teachers	First- and second-grade classrooms
Keeping Foster and Kin Parents Trained and Supported (KEEP)/Chamberlain [70]	Program promotes child well-being and prevents foster placement breakdowns through support and skill enhancement of foster and kinship parents. The program has demonstrated positive outcomes for treatment and prevention of child and adolescent behavior problems in multiple randomized control trials [70–72] and significant reductions in marijuana, tobacco, and other drugs at 18 months [73] as well as sexual behavior [74, 75].	Middle school	Research: randomized implementation trial^a^ Practice: full scale implementation in New York city through child welfare system; active implementation^b^	Foster/kin parents supported by trained health workers	Foster/kin parents groups
Life Skills Training (LST)/Botvin, Griffin [76]	Prevention of substance abuse (alcohol, tobacco, drug use) and violence. Program resulted in significant, long-lasting reduction in drug use [77, 78] and HIV risk behaviors [79].	Elementary, middle, and high school	Research: randomized implementation trial^a^ Practice: implementation in schools through the National Health Promotion Associates (http://www.lifeskillstraining.com/index.php); sustainment^b^	Trained school personnel	School classrooms
Nurse-Family Partnership/Olds [29]	Increase pre-natal and early stage parenting skills. The program resulted in significant reductions in youth alcohol use, fewer sexual partners, and fewer problems with alcohol or drugs in a 15-year follow-up [80].	First 2 years of life	Research: natural experiment, type 1 hybrid design^a^ Practice: implementation in communities through the NFP National Service Office; sustainment^b^	Trained nurses	Home visits
Sisters Informing Sisters about Topics on AIDS (SiSTA)/Wingood, DiClemente [81]	Demonstrated high increases in condom use and when combined with a biological intervention of HPV vaccination, demonstrated significant reductions in incident high-risk HPV infection [82]	Young adulthood and adulthood	Research: type 1 hybrid design^a^ Practice: wide scale implementation through CDC’s Dissemination of Evidence-Based Intervention (DEBI) program (http://www.effectiveinterventions.org/en/HighImpactPrevention/Interventions/SISTA.aspx); active implementation^b^	Peer/near peer facilitator and health educators	Community-based setting
Strong African American Families Program (SAAF)/Murry, Brody [32, 83]	Improve parent-child relationships. Significant preventive effects in initiation of alcohol use and sex risk behaviors [84] through improved parent-child relationships [85, 86].	Middle school, high school	Research: type 2 hybrid design^a^ Practice: exploration phase^b^	Caregivers supported by trained facilitators	Community-based settings, community churches

^a^[70]

^b^[15]

Implementation of these programs currently ranges from some programs being completely unused outside of a research setting to other programs being rolled out at the national, state, and community level at a fast pace. For example, Familias Unidas [26], a parent-centered preventive intervention for Hispanic parents and their adolescents, is currently available only through the developers in Miami whereas Life Skills Training (LST), a primary prevention program for adolescent drug abuse, delivered in middle schools, has the broad distribution, being used in 10,000 US schools in every US state and in 32 counties [27]. The Good Behavior Game (GBG), a universal program targeting substance abuse, for first and second grade students [10, 11], also has broad distribution and is currently being implemented in 29 school districts through funding from the Substance Abuse and Mental Health Services Administration (SAMHSA), and a randomized implementation trial of two training/supervision strategies is underway [28].

The Nurse-Family Partnership program, which starts prenatally and continues through the first 2 years of life, has been implemented in more than 440 counties in the USA and served more than 26,000 families, and its implementation strategy [29] is being updated through a non-experimental continuous quality improvement approach [30, 31].

The effectiveness of the Strong African American Families (SAAF) program, designed to prevent the initiation and escalation of alcohol use, among rural African American families with middle school children, when delivered by a community provider, was recently evaluated in a randomized trial with public school children across 8 counties of rural Georgia [32].

At the current time, most of the prevention programs have implementation strategies based on each program’s unique needs rather than being informed by studies of implementation. Our only exception to this is the implementation model of the Communities That Care (CTC) [33], where the community can choose to determine what prevention programs best suit their needs. Thus, CTC is a general implementation strategy not specific to any particular intervention.

To understand how the implementation strategies relate to one another, a myriad of implementation frameworks have been put forward for behavioral interventions [34]. It is often difficult to map the sub-dimensions of these implementation frameworks into specific strategies, so in this paper, we have taken a different, fundamentally engineering approach to characterizing implementation systems. By *characterizing* implementation strategies across different levels of system influence, we can represent their different components and ultimately redesign the system to respond more efficiently to its environment, resources, and objectives.

### Systems engineering applied to implementation research

Systems engineering is both a discipline and process to guide the development, implementation, and evaluation of complex systems in order for the success of the system to be realized [35]. Systems engineering has a holistic focus and attempts to ensure all aspects of a system are considered and integrated into a whole. Thus, this approach is not only concerned with the design of the elements or components of a system but also with external factors that can constrain the design such as logistical support requirements, individual needs, and available resources [36].

System thinking and general systems theory dates back to Bertalanffy in the 1930s and the systems approach was developed initially in the biological sciences and refined in the 1940s in the communication and military industries [37]. The systems approach has evolved throughout the years because of the increased complexity of systems largely due to continual developments in technology. The concepts and methods of systems engineering have been applied to enhance the performance of aviation, manufacturing, logistical, aerospace, and medical systems. In recent years, a thrust has been on human-machine systems. Within the discipline of human factors engineering, a human-machine system is composed of four main interdependent components; the human, the task/activity they are performing, the equipment/technology they are using to perform this task, and the context/environment (physical and organizational) where this transaction is occurring.

Recently, the National Academy of Engineering (NAE) and the Institute of Medicine (IOM) issued a report calling for the application of systems engineering methods to healthcare delivery systems and improved collaboration between medicine and engineering in healthcare delivery [38]. To help clarify the complexity of the healthcare system, the report adopted a four-level model: (1) the individual patient; (2) the care team; (3) the organization; and (4) the social and political environment. The report underscored that optimizing the system as a whole requires of moving away from “silos” thinking and instead adopting “systems thinking”, which recognizes the interdependence among the levels of the model. Following this report, Tu and colleagues [39] examined the feasibility of applying systems engineering techniques used in traditional settings such as manufacturing, to implement an evidence-based intervention, a colorectal screening protocol, at a community health center. They found that the application of these techniques in healthcare settings is feasible but that there are unique challenges related to patient and organizational factors. They also stress that more research is needed to guide the application of these approaches in healthcare environments.

**Fig. 2:**
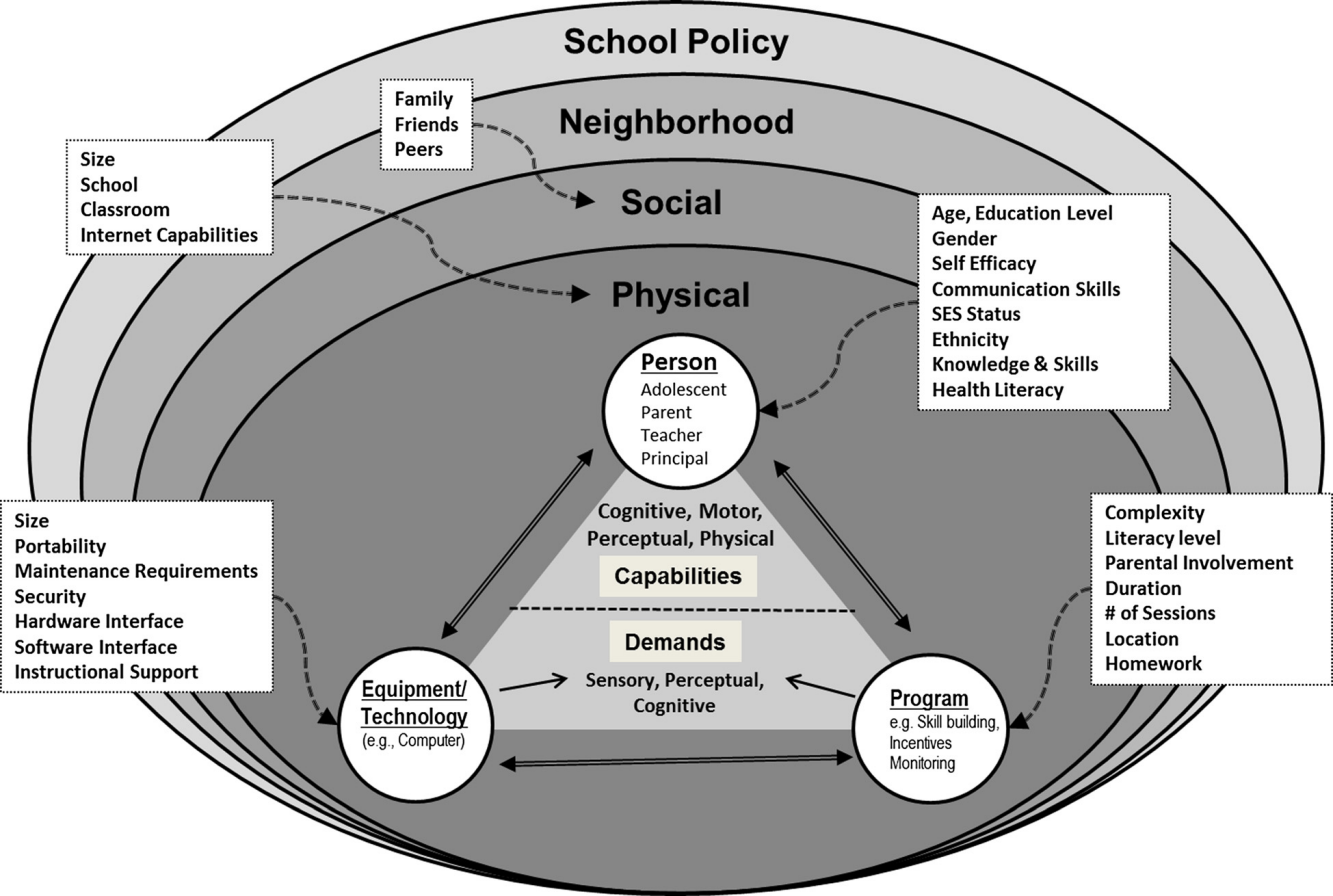
Systems model of a drug abuse prevention program [87]

Generally speaking, a system can be conceived as an aggregation of components organized according to some structure to accomplish a set of goals or objectives [37]. Using a “systems framework,” within the domain of intervention research, a prevention program can also be conceptualized as a complex system with components that interact to achieve specific goals and objectives. For example, a drug abuse prevention program that has the overall objective of reducing and or preventing the use of illegal substances among high school students can be conceived of as a complex system with interacting components. At a macro level, these components include the intervention, the mechanisms of action (e.g., skill building exercises), the people who deliver the intervention (intervention agents), the target population, and the place where the intervention is delivered. This system also operates within a larger environment and includes the school, school district, and neighborhood. The child’s environment also includes home, peer, community (including drug-related norms, accessibility, and HIV viral load), and virtual environments (Fig. 2). All of these components have varying and dynamic characteristics and interact with each other.

From a systems engineering perspective, optimization of successful implementation of a prevention program in a community setting requires that all of the components of an intervention system be considered in the implementation process. For example, implementation of a drug abuse prevention program requires an understanding of the school’s resources, policies, and readiness for change; the community/neighborhood of the children and the characteristics of those children; and their family situations. These factors all influence the degree to which intervention fidelity is likely and whether the goal of reduced substance abuse can be achieved. If the school has limited resources and the intervention is too burdensome with respect to the number and scheduling of sessions and reporting requirements for teachers, it is likely to be implemented poorly and less likely to be effective.

Thus, from a systems perspective, a fundamental aspect of successful implementation of an intervention within a community setting requires an understanding of the characteristics and associated implementation demands associated with the intervention (e.g., if the intervention requires Internet access; or 9 weekly 60-min sessions and 5 parent support group sessions) as well as the characteristics and constraints associated with each of the other components of the “intervention system” such as the target population and the organization, environment/setting where the intervention will be implemented.

Wandersman and colleagues [40] also advocate the use of a systems approach to help bridge the gap between prevention research and practice. They propose a framework, the Interactive Systems Framework (ISF) for Dissemination and Implementation. The framework focuses on how to move evidence-based prevention programs into practice and not on the development and testing of new interventions, which is a central component of our approach. However, the ISF does underscore the importance of considering the characteristics of the organization and the interdependencies among the components of the system. Greenhalgh and colleagues [41] also propose a systems model, based on a systematic review of health service innovations to guide the dissemination and implementation of innovations. The model takes into account various system components (innovation, organization, adopter) and some of the characteristics of these components such as the technical knowledge requirements of the innovation, organization readiness for innovation, and the adopter’s skills and motivation. However, as noted by the authors, the model is intended mainly as memory aid for considering the different aspect of a complex situation and their interactions and should not be viewed as a prescriptive formula. In addition, some of the component characteristics that are listed are rather general (e.g., “fuzzy boundaries” of the innovation) and may be difficult for an intervention researcher to operationalize.

### Data collection protocol

The data collection protocol had two complimentary components, a survey that gathered information regarding the basic characteristics of the 10 evidence-based prevention programs and a guided telephone interview. The focus of the survey was on gathering information on the essential elements of the programs such as delivery characteristics, staffing, and degree of program adaptability. The telephone interview was intended to compliment the survey and allowed us to explore program implementation in greater depth and addressed issues related to the implementation context, training, intervention fidelity, community support and perceived implementation challenges. To insure that we had a basic understanding of the programs before conducting the interviews, the interviews were conducted after the survey was completed. To provide a context for the interview, the interviewees were provided with a hand out that briefly explained the systems engineering approach.

### The implementation survey

The survey was adapted from the Intervention Taxonomy (ITAX), a survey instrument designed to help interventionists describe and understand the essential features of an intervention protocol [42]. The survey contained 45 items which were grouped into six sections: (1) background information (e.g., number and type of intervention components such as initial component, maintenance component, home component, classroom component; identifying information for the person completing the survey), (2) program characteristics, (3) measurement of goals and processes, (4) staffing and training, (5) cost analysis, and (6) partnerships/foundations. The section on program characteristics included items that captured the following information: mode of intervention delivery (e.g., face-to-face, online), materials used (e.g., pamphlets, videos), setting of delivery (e.g., classroom, clinic, home), intervention strategies (e.g., problem solving, skill building), intervention schedule (e.g., duration of program, number of sessions), and adaptability (e.g., type of adaptability (language) and processes for adaptability). The section on measurement of goals and processes included three items related to the types of outcomes being assessed, the assessment schedule, and who conducted the assessments. The staffing and training section contained items related to characterizing the skills and qualification requirements of the people involved in the delivery of the intervention and type of and intensity of staff training. The section on cost analysis contained items related to variables that could be included in a cost analysis and the schedule for assessing these variables. The section on partnerships/foundations included items related to types and importance of community partnerships and the functions of the Community Advisory Board (if applicable).

For ease of administration, many of the item responses were multiple choice. The survey was completed online. In most cases, it was initially completed by a Project Manager/Coordinator and the Principal Investigator (PI) reviewed the responses and resolved any discrepancies.

### The interview

The interview complemented the survey and enabled participants to describe their program in more detail. For example, more detailed information was gathered on the target population (e.g., skill level; SES status), the characteristics of the community/setting and all of the “players” involved in program implementation and so on. Issues of training and intervention fidelity were also probed in greater detail. In addition, information was gathered on perceptions of factors important to the success of the program and challenges to its implementation. A semi-structured format was used to guide the interview. It was conducted via teleconference and the duration was 60–90 min. The interviewees included the PIs of the programs. In some cases, it also included program staff such as a Project Coordinator.

The interviews were conducted by a senior researcher from a different field than drug/HIV prevention (SC), who was deliberately kept naïve to detailed scientific literature on each of these programs. Thus, the only information available prior to conducting these interviews consisted of the answers to the survey questions. The survey and interview protocol are available from the first author.

## Results

The data gathered in this study elucidate the characteristics of the 10 prevention programs that were in various states of implementation. The objective is to demonstrate how a systems engineering approach could be used to identify the requirements for implementing these programs in a community setting. The study was also designed to identify essential elements of and potential barriers to successful implementation of these programs. We first describe the program characteristics gathered from the survey. We then report on the findings from the interviews with the Principle Investigators. The interviews were transcribed and the topics that emerged were summarized into two main categories: (1) factors important to successful implementation and (2) challenges to program implementation.

### Characteristics of the 10 programs

As shown in Table 2, there was considerable variability across the programs with respect to intervention schedule. However, on average, the duration of most programs is about 22 weeks and the average number of sessions is 25 with an average contact time per session of about 56 min.

**Table 2:** Summary of preventive intervention schedule characteristics

		Duration (weeks)	Number of sessions	Contact time (minutes/session)
*N*	Valid	14	14	14
	Missing	1	1	1
Mean		22	25.07	56.58
Median		11	9	60
Standard deviation		33.911	16.276	32.870
Range		128.0	63	119
Percentiles	25	5.500	4.000	45
	50	12.000	9.000	60
	75	33.911	15.750	82.500

This table does not include the implementation strategy of Communities That Care (CTC), which consists of 5 required sessions and 1 optional session (4.5 days over 2-year period), and the implementation support for the Good Behavior Game (120 weeks, 2 days a week)

All of the programs include face-to-face delivery of the intervention but varied considerably on the use of other media. They also vary on the behavioral intervention approach. All of them involve the use of skill building exercises and almost all involved provision of information and use of problem solving techniques. Most provided tracking and monitoring of behavior (64 %) and most (55 %) provided incentives, didactic instruction, and stress management techniques. Intervention delivery location was also quite varied with no location being predominant (Table 3).

**Table 3:** Summary of program delivery characteristics and features of adaptability

	*N*	Percent
Program delivery characteristics		
Mode of intervention delivery		
Face-to-face contact	11	100 %
Telephone contact	4	36 %
Computer/Internet contact	3	27 %
Video/CD	4	36 %
Print media	5	46 %
Lectures	2	18 %
Technology (computer or telephone)	4	36 %
Type of intervention strategy		
Provision of social support	5	46 %
Tracking and monitoring	7	64 %
Provision of information	10	91 %
Provision of behavioral incentives	6	55 %
Didactic instruction	6	55 %
Skill building techniques	11	100 %
Problem solving techniques	10	91 %
Stress management techniques	6	55 %
Other techniques	5	46 %
Intervention delivery location		
Participant home	5	46 %
Classroom	4	36 %
Physician’s office	.	.
Hospital/clinic	2	18 %
Work site	.	.
Community center	5	46 %
Nursing home	.	.
Group residential facility	.	.
Clinical research space	3	27 %
Types of materials used		
Information sheets/checklists/pamphlets	7	64 %
Manuals/workbooks	11	100 %
Internet	4	36 %
Video	8	73 %
Audio	3	27 %
Live demonstrations	7	64 %
CDs/DVDs	5	46 %
Other Materials	1	9 %
Program adaptability features		
Intervention strategies are adapted for cultural sensitivity	11	100 %
The program is scripted	11	100 %
Degree of scripting		
Minimal guidelines	1	9 %
Goals of each session are specified but no further scripting	3	27 %
Goals and exercises/tasks of each session are specified	6	55 %
Specific language is provided, with room for elaboration	8	73 %
Exact scripts are provided for humans to speak	2	18 %
Intervention is delivered by machine	.	.
Other	2	18 %
Aspect of intervention amenable to adaptation		
Number of sessions	4	40 %
Schedule of sessions	6	60 %
Duration of sessions	4	40 %
Location of intervention delivery	4	40 %
Focus of sessions	3	30 %
Intervention scripts	1	10 %
Determination of modifications		
Clinical judgment	4	36 %
Formal checklist/interventionist	3	27 %
Intervention MOP	3	27 %
Participant choice	3	27 %
Computerized algorithm	.	.
Other determination	5	46 %
Time of modifications		
At intake/initial assessment	2	18 %
At scheduled increments	2	18 %
At any session/contact	4	36 %
At other point	4	36 %

The protocols for all of the programs are scripted but all programs also incorporate protocols to adapt some aspects of the intervention, such as the schedule, duration, or number of sessions or the session delivery location. Most programs (8) include specific guidance on how to adapt the program and to provide exact scripts for program adaptation. The impetus for the adaptation varies and includes clinical judgment and requests from participants. The programs can also be adapted to be responsive to the needs of different cultures, for example, involving members of the participants’ community in recruitment; or including individuals from a target organization (e.g. school) in the delivery of the intervention. A small number of programs (4) include interventionists whose racial/ethnic background is matched to that of the program participants. For most programs (9), the content of the intervention strategies was developed to match the target population’s literacy level.

The research teams use a variety of types of staff to deliver the programs including the following: assessors (*n* = 5), social workers or counselors (*n* = 4), nurses (*n* = 2), teachers (*n* = 2), and physicians (n = 1). This varied according to the needs of the program. For example, the Nurse-Family Partnership program involves home visits to mothers by public health nurses. For all programs, the staff receives specific training on the intervention. Interestingly, four of the programs require staff to speak a language other than English, such as Spanish, to accommodate the needs of the target population and the community.

About half of the programs gather data related to program goals (e.g., alcohol abuse, tobacco use) pre and post implementation and others gather this information at several times points (e.g., monthly). Common program goals include changes in behavior, enhanced behavioral and problem solving skills and increased knowledge. Data collection methods vary and generally include the use of questionnaires and rating scales, objective measurement, and interviews.

Finally, when asked about partnership building, surprisingly only 20 % of the programs have an advisory board. Those that do have boards indicated that the board functions to review program protocols, procedures, and measures and advises on implementation strategies. Most of the respondents (80 %) indicate that political support of their program was important to implementation.

### Factors important to successful program implementation

Several main themes emerged during the interviews regarding factors important to facilitating successful program implementation. First is the importance of having a trusted, collaborative partnership with community groups/agencies and personnel within the targeted setting for program implementation. The general perception of the investigators is that implementation success is highly dependent on the extent to which the program team has a deep and lasting relationship with the community-based organizations (CBOs) or institutions that would ultimately be responsible for program implementation. Several investigators specifically mentioned the importance of a Community Advisory Board (CAB). For the programs included in this study, these partnerships were generally between academically based program teams composed of the program creators, research coordinators, and evaluators with community and agency leaders and staff. They also involved healthcare clinics, school districts schools, churches, coalitions, and law enforcement agencies. Furthermore, they were typically multi-level and involved numerous “players” including physicians, teachers, parents, and in some cases custodial and security personnel. As one interviewee said “A lot of how we [researcher and research team] did what we did was based on… these people [community partners] telling us what it is that we would need to do in order to make this [research] work, and we listened to them… you [researcher] have to be inclusive and you have to find ways to make people feel that you’re there not just to get from them but to provide as well.”

Second, partner agencies need to have a strong commitment to the program. Most of the interviewees felt strongly that if their partner agencies were not committed to the program and did not remain committed, implementation would fail. This commitment entails several elements. One essential element is that the agency has to have a complete understanding of the program’s characteristics and requirements. A second component is the agency’s readiness to implement the program and a third is having the staff, resources, and support necessary for a program. Another essential element is agency “buy-in”; the degree to which the agency is willing to expend the effort needed to take ownership of the program. This might include, for example, reducing the workload of case workers so that they have sufficient time to deliver the program or adapting the number of sessions to accommodate families. One interviewee discussed a reason for lack of receptivity among parents for a family-based program was the lengthy duration of the program. “Unable to commit to a 7-week program because of their time schedules or they had children in developmental situations … working swing shift jobs, those kinds of things.” Another key element of “buy-in” is having staff engaged in and able to deliver the program. The skill level of the staff is also critical to program success. In addition, if a program includes group activities the cohesiveness of the group is important. Many of the interviewees also mentioned the importance of flexibility. Successful implementation often depended on research staff being able to modify and adapt the program to local needs and exigencies. This might include adapting the language and exemplars used in a program or adapting a program to be delivered in a particular setting such as a church as opposed to a clinic or to the structure or personnel within an organization. For example, as one interviewee responded: “I think we are going to have to be more flexible in terms of fidelity because of skill level… let’s say there’s one principal for three middle schools and the person who has the most contact with the parents by default is the secretary in the front office … because of her skill set … we have to take out or redo some of the demands of our program.”

### Common challenges to successful implementation

One major challenge identified by most of the interviewees is having strategies to assess and manage treatment fidelity. In some cases assessment of intervention fidelity is resource intensive and time-consuming. Not surprisingly it is also difficult to maintain fidelity over time. Most of the respondents also indicated the need for strategies for mechanisms to implement “corrective action” if required. As expected, there is tension between program fidelity and flexibility. As noted, flexibility or the ability to adapt a program to cultural or contextual nuances is often a key to successful implementation yet this adaption also raises concerns that core elements of the program might be omitted or changed in ways that reduce the effectiveness of an intervention.

An additional challenge to implementation for many programs is scheduling and logistics. Most of the interviewees stated that they faced challenges scheduling program sessions or lesson delivery. Collaborating agencies often have many demands that outweigh the desire to implement an intervention program. Scheduling and logistics challenges can be exacerbated by staff turnover and/or promotion both within the program development team and within partner CBOs is a critical challenge. Furthermore, the logistics associated with training staff when the program is being implemented at multiple locations—including international settings—is also a challenge and often resource demanding. Overall, the interviewees were conscious of the challenge of coordinating these activities and of attending to the many logistical challenges sustainable programs require.

In sum, adequate resources within CBOs and allocating these resources to program implementation are a major concern and represent a continued challenge to the implementation of intervention programs. Another major challenge is securing a commitment on the part of the organization to implement the program in a way that is consistent with program requirements.

## Discussion 

The goal of this paper was to demonstrate how a systems engineering approach could be used to identify the requirements for implementing prevention programs, focused on the prevention of drug or HIV sex risk behaviors, in a community setting. The study was also designed to identify factors important to and potential barriers to implementation success. We provide data from a survey and interview with 10 evidence-based prevention programs that were in various states of program implementation.

In general, the programs were quite varied in terms of their characteristics and implementation requirements and strategies. Several important themes emerged that cut across all programs with respect to successful implementation, however. First, the tension between fidelity and adaptability is high. All of the programs were scripted and contained core elements, yet adaptation of a program is generally required to meet community needs and shifting resources and priorities. In addressing this tension, the community-research partnerships arrived at different decisions regarding adaptation, with respect to what aspects of the protocol can be adapted, to what extent and by whom. We recommend that during the design and evaluation of interventions researchers pay special attention to what aspects of an intervention can be adapted and provide general guidelines or protocols for implementing and documenting adaptations both during research trials and in practice settings. Adaptation issues should also be addressed in CONSORT-type checklists for randomized intervention and implementation trials.

Also, all of the study participants recognized the need to maintain program fidelity but struggled with ways to measure and maintain this fidelity given community constraints. From a systems perspective, simply monitoring for fidelity is not sufficient to maintain standards; mechanisms for feedback and accountability also need to be in place [37]. It is important to measure the extent to which the intervention was implemented as intended, which, if low, could threaten internal validity and ultimately external validity. Thus, another important dimension to capture in intervention protocols is a description of monitoring and feedback subsystems, including who is ultimately responsible for overseeing that the program is delivered with fidelity. Coupled with this challenge is monitoring the implementation strategy itself. Having a written protocol of what stages and steps are needed for implementation, along with a mechanism to track protocol violations, is also important to include in an intervention Manual of Operations (MOP) and in CONSORT-type checklists for implementation trials much like the specialized one that now exists for cluster randomized trials [43].

All of the study participants also emphasized the need for a strong and committed partnership between the program developers, implementation researchers, and the community partners who will be involved in implementing the program. In this respect, many of the programs included in this study relied on a community partner such as a school, church, coalition, or other agency to help with access to clients and communities; and to help with program delivery. In all cases, the commitment of these partners is considered essential to program success. Also, having a strong partnership is critical to ensuring effective communication between researchers and organizational staff. Determining the optimal structures and functioning of community coalitions is an active area of research [44, 45], and information regarding these issues should also be provided in an intervention MOP.

The factors identified through the interview and survey presented above are also supported by the dissemination and implementation conceptual model put forth by Aarons and colleagues [15], which divides factors affecting implementation into two contexts, the *outer context* (i.e., community advisory board) and the *inner context* (i.e., agency “buy-in”, agency readiness, agency flexibility). Like Aarons and colleagues [15] suggest, our interviewees concluded that the importance of the inner and outer contexts varies depending on the implementation phase of the program.

## Conclusions

This paper has identified a number of implementation challenges. However, these implementation challenges can also be considered opportunities for implementation science. For example, they imply that better tracking and coordination systems could help reduce the costs and barriers to successful implementation. The identified challenges also indicate that a process to help determine how to implement a program to achieve the optimal balance of maintaining program fidelity and adaptability to meet contextual demands would be a useful next step for implementation science. The use of technology systems to facilitate staff training especially staff in remote locations could also prove to be useful.

The systems perspective has enabled us to highlight these challenges and demonstrate how comprehensive descriptions of interventions facilitate understanding the requirements of program implementation and decisions about the feasibility of implementing a program in community settings. Intervention researchers in collaboration with community service providers can determine if they have adequate staff and resources to successfully replicate the essential elements of a program and if the program is sensitive to and meets the “culture” of the community. Indeed, a “cost calculator” tool already exists in child welfare that helps organizations and communities budget and plan for how a specific program can be implemented in their settings [46]. This systems engineering approach could lead to a toolkit for communities and researchers to examine all required resources for an intervention, not just ones with monetary value. The tools used in the approach can also be used to evaluate different implementation strategies whether they take place in randomized implementation trials or adaptive [23] or hybrid trials [47] that combine effectiveness and implementation.

However, it should be noted that the usefulness of this approach depends on having a common language/taxonomy for describing programs and implementation strategies. This is a challenge within implementation science. Currently, despite some attempts, there is as yet no fully accepted standard language/taxonomy for describing the components and processes of prevention delivery systems across diverse fields [40, 48–50] or for implementation designs. Additional work is required to distinguish between programs in local investigations and implementation research [51], as well as to distinguish the current mélange of overlapping terms for trial designs in this field [18, 23, 52]. This makes it difficult to tease out the key elements of a program and to systematically conduct cross-study analyses. For example, in the development of the ITAX survey instrument that was applied to interventions that targeted healthy behaviors, a consensus process was used to identify intervention elements relevant to understanding outcomes and important to subsequent replication efforts. In addition, operational definitions of the terms used in the survey were provided and pilot tested with a sample of the investigators. This provided some assurance that the survey respondents were “speaking the same language.” This is an area that needs more work within implementation science as terms such as “programs,” “treatment,” and “implementation strategy” can take on different meanings.

Another challenge for implementation science is to identify strategies that can address implementation challenges so that program implementation proceeds more efficiently. This will almost certainly involve innovative uses of mixed methods [53] and technology (e.g., to assist with training across implementation sites) or systems science methods [13, 54, 55], as well as improved decision-making, especially with interventions that are rapidly evolving [56] or providing of a rich set of options at the individual and community level. Moreover, such strategies will be needed to create more effective and sustainable programs in the future especially as many of the programs described in this study are being implemented on a broad scale across diverse communities and cultures, including populations where health disparities are greatest [13, 20].

This survey and interview were tested together on 10 prevention programs for drug abuse and/or HIV sex risk behavior prevention, and thus we are not able at this time to generalize to their value in other settings, including treatment in contrast to prevention, where randomized implementation trials are being designed. However, the breadth of these 10 programs, and the deliberate choice of having the interviews conducted by a researcher who was unfamiliar with these particular programs and this particular scientific field, suggests optimism that this approach could be valuable for not only specifying differences in a wide variety of randomized implementation trials but also for purposes of training new investigators and providing useful feedback to the researchers and community leaders themselves. Other researchers conducting intervention trials can use this approach to help determine the essential requirements of their intervention, which will provide information about requirements for broad scale implementation.

However, this study has some limitations. As noted, it was restricted to trials concerned with prevention of substance abuse and sexual risky behaviors. Thus, it needs to be replicated with other types of intervention programs. In addition, we only examined limited components of the “intervention system” from the perspective of the researcher. To fully gain benefits from this approach, a more detailed analysis of the characteristics of the target population and other individuals involved in implementing the intervention should be conducted. If for example, the intervention is being delivered in a school setting and involves parents and students, an analysis of the characteristics of the students, parents, and teachers would be helpful to understand their preferences, skills, attitudes, and needs. Additionally, an analysis of the context should be conducted including the organization (e.g. school) as well as the neighborhood and larger social and political context. Finally, other aspects of the intervention should be included in the analysis such as the usability, perceived value, work flow, and communication patterns. The ultimate goal of the systems approach is to identify potential mismatches or degree of fit between the elements and requirements of an intervention; the needs, preferences, and characteristics of the target population; and the characteristics and resources/constraints of the implementation setting.

## Footnotes

1 We also conducted a survey of the SUCCESS prevention program, aimed at reducing cervical cancer in Haitian women; results for this study are not included in this paper because the intervention target differs from the other 10.
